# The Compartmentalized Bacteria of the Planctomycetes-Verrucomicrobia-Chlamydiae Superphylum Have Membrane Coat-Like Proteins

**DOI:** 10.1371/journal.pbio.1000281

**Published:** 2010-01-19

**Authors:** Rachel Santarella-Mellwig, Josef Franke, Andreas Jaedicke, Matyas Gorjanacz, Ulrike Bauer, Aidan Budd, Iain W. Mattaj, Damien P. Devos

**Affiliations:** 1 European Molecular Biology Laboratory, Heidelberg, Germany; 2 Laboratory of Cellular and Structural Biology, The Rockefeller University, New York, New York, United States of America; The Scripps Research Institute, United States of America

## Abstract

The development of the endomembrane system was a major step in eukaryotic evolution. Membrane coats, which exhibit a unique arrangement of β-propeller and α-helical repeat domains, play key roles in shaping eukaryotic membranes. Such proteins are likely to have been present in the ancestral eukaryote but cannot be detected in prokaryotes using sequence-only searches. We have used a structure-based detection protocol to search all proteomes for proteins with this domain architecture. Apart from the eukaryotes, we identified this protein architecture only in the Planctomycetes-Verrucomicrobia-Chlamydiae (PVC) bacterial superphylum, many members of which share a compartmentalized cell plan. We determined that one such protein is partly localized at the membranes of vesicles formed inside the cells in the planctomycete *Gemmata obscuriglobus*. Our results demonstrate similarities between bacterial and eukaryotic compartmentalization machinery, suggesting that the bacterial PVC superphylum contributed significantly to eukaryogenesis.

## Abstract 2

Compartmentalized bacteria have proteins that are structurally related to eukaryotic membrane coats, and one of these proteins localizes at the membrane of vesicles formed inside bacterial cells.

## Author Summary

Despite decades of research, the origin of eukaryotic cells remains an unsolved issue. The endomembrane system defines the eukaryotic cell, and its origin is linked to that of eukaryotes. A search was conducted within all known sequences for proteins that are characteristic of the eukaryotic endomembrane system, using a combination of fold types that is uniquely found in the membrane coat proteins. Outside eukaryotes, such proteins were solely found in the Planctomycetes-Verrucomicrobia-Chlamydiae (PVC) bacterial superphylum. By immuno-electron microscopy, one of these bacterial proteins was found to localize adjacent to the membranes of vesicles found within the cells of one member of the PVC superphylum. Thus, there appear to be similarities between bacterial and eukaryotic compartmentalization systems, suggesting that the bacterial PVC superphylum may have contributed significantly to eukaryogenesis.

## Introduction

Eukaryotic cells are subdivided into membrane-bound compartments with specialized functions. The exchange of material between these compartments and between the inside and outside of the cell is essential to maintain cellular integrity. Exchange is mediated by membraneous vesicles budding from a donor membrane and fusing with a target one, either on one of the compartments or the plasma membrane. Vesicle budding is initiated by the polymerization of a protein coat that ultimately surrounds the membrane vesicles. Membrane coat (MC) proteins are key to this process since, in combination with their adaptors and regulators, they are sufficient to induce coated vesicle formation [1]. MCs are essential. The identity of the MCs defines the three classes of coated vesicles: clathrin in clathrin coated vesicles, α- and β'-COP in coat protein complex I (COPI) vesicles, and Sec31 in COPII vesicles [2]. It is now well supported that all MCs are related and that this homologous relationship can be extended to some of the nucleoporins that form the nuclear pore complex, which allows selective transport across the nuclear envelope. This relationship is based on a unique combination of protein domains that is exclusively found in all eukaryotic MCs. This hypothesis was initially based on protein structure predictions [3] but has since been supported by structural studies of vesicle and nucleoporin MCs [4]–[12]. In structural biology, a protein architecture describes the type, number, and order of domains composing a protein. The MC architecture consists of an amino-terminal β-propeller domain followed by a carboxy-terminal Stacked Pairs of α-Helices (SPAH; also referred to as α-solenoid) domain. β-propeller domains are formed by six to eight β-blades, each blade composed of four β-strands, arranged circularly around a central axis. SPAH domains consist of pairs of α-helices stacked on each other in a more or less linear fashion. β-propeller and SPAH domains are present in the proteome of all organisms. However, their combination in this particular architecture has so far only been found in eukaryotic MCs [3],[13], in a subset of the proteins forming the coats around budding vesicle (e.g., yeast clathrin or Sec31), and the pores in the nuclear envelope (e.g., Nup120). Despite the evidence for the common ancestry of the MCs, their origin, and the one of the eukaryotic endomembrane system, is still unknown. However, because of their central role in eukaryotic cell organization, and as sequence- and structure-based searches have shown that the endomembrane system was already complex in early eukaryotes [14],[15], MCs are expected to be present in the most recent eukaryotic common ancestor. No prokaryotic MC homologues are detectable by sequence homology searches [3]. As structure is more conserved than sequence during the course of evolution, we used structure prediction [3],[13] to search for additional proteins with the MC architecture.

## Results

We searched 687,835 eubacterial proteins in 162 complete and 13 incomplete proteomes, 60,382 archaebacterial proteins in 27 complete proteomes, and 231,229 eukaryotic proteins in 23 complete proteomes, totaling 979,446 screened proteins in 212 complete and 13 incomplete proteomes (Tables S1 and S2). Since we aimed at maximizing the sensitivity of detection, we used one of the most sensitive tools [16] with a permissive cut-off. Our final fold predictions (Table 1) were evaluated and are supported by several considerations [3],[13], including fold assignment program scores, secondary structure prediction agreement, atomic model evaluation by statistical potential (Table 2, Text S1), and, for a selected protein, limited proteolysis (see below).

**Table 1:** Number of membrane coat-like proteins in the PVC superphylum.

Species	Phylum	Genome Status	Total Number of Proteins	Number of MCs
*Chlamydophila felis* Fe/C-56	C	F	1,013	0
*Candidatus Protochlamydia amoebophila* UWE25	C	F	2,031	0
*Chlamydia muridarum Nigg*	C	F	911	0
*Victivallis vadensis* BAA-548	L	D	3,541	0
*Lentisphaera araneosa* HTCC2155	L	D	5,104	9
*Candidatus Kuenenia stuttgartiensis*	P	F	4,663	0
*Blastopirellula marina* DSM 3645	P	D	6,025	11
*Planctomyces maris* DSM 8797	P	D	6,480	11
*Rhodopirellula baltica* SH 1	P	F	7,325	5
*Gemmata obscuriglobus* UQM 2246	P	D	7,989	8
*Akkermansia muciniphila* BAA-835	V	F	2,176	0
*Methylacidiphilum infernorum* V4	V	F	2,462	0
*Opitutaceae bacterium* TAV2	V	D	4,036	0
*Opitutus terrae* PB90-1	V	F	4,632	0
*Pedosphaera parvula* Ellin514	V	D	6,402	9
*Verrucomicrobium spinosum*	V	D	6,509	16
*Chthoniobacter flavus* Ellin428	V	D	6,716	14

V, Verrucomicrobia; L, Lentisphaerae; P, Planctomycetes; C, Chlamydiae; D, draft; F, finished.

**Table 2:** *G. obscuriglobus* proteins have the MC architecture.

Identifier	Size^a^	Modeled Fragment Length	Modeled Fragment Location^a^	Fold	Z-score^b^
4750	1,287	384	Nt	β-propeller	−6.9
4750	1,287	273	Ct	SPAH	−8.9
4796	1,039	326	Nt	β-propeller	−3.7
4796	1,039	188	Ct	SPAH	−5.5
4978	1,158	362	Nt	β-propeller	−7.2
4978	1,158	277	Ct	SPAH	−8.8

Model and protein information are provided for three representative *G. obscuriglobus* MC proteins.

a Protein size and fragment location: because the initiation codon is unknown, the size of the proteins is only indicative of the maximum and we only locate the domains relative to each other. Nt, amino-terminal; Ct, carboxy-terminal.

b Z-score [56] of the comparative model for the modeled fragment. A Z-score below −3 is considered to be highly significant.

At least four MCs are expected to be found in each eukaryotic proteomes, corresponding to clathrin, Sec31, the pair of homologues α- and β'-COP, and one nucleoporin. We found at least four MCs proteins in most eukaryotes, with a few exceptions, like *Plasmodium falciparum*, where we found only two. This might be explained by our failure to detect all MCs in this organism but is perhaps more likely to be due to the peculiar cellular biology of this organism, given that in all other eukaryotes, our method recovered at least one copy of all four groups of MCs.

**Figure 1:**
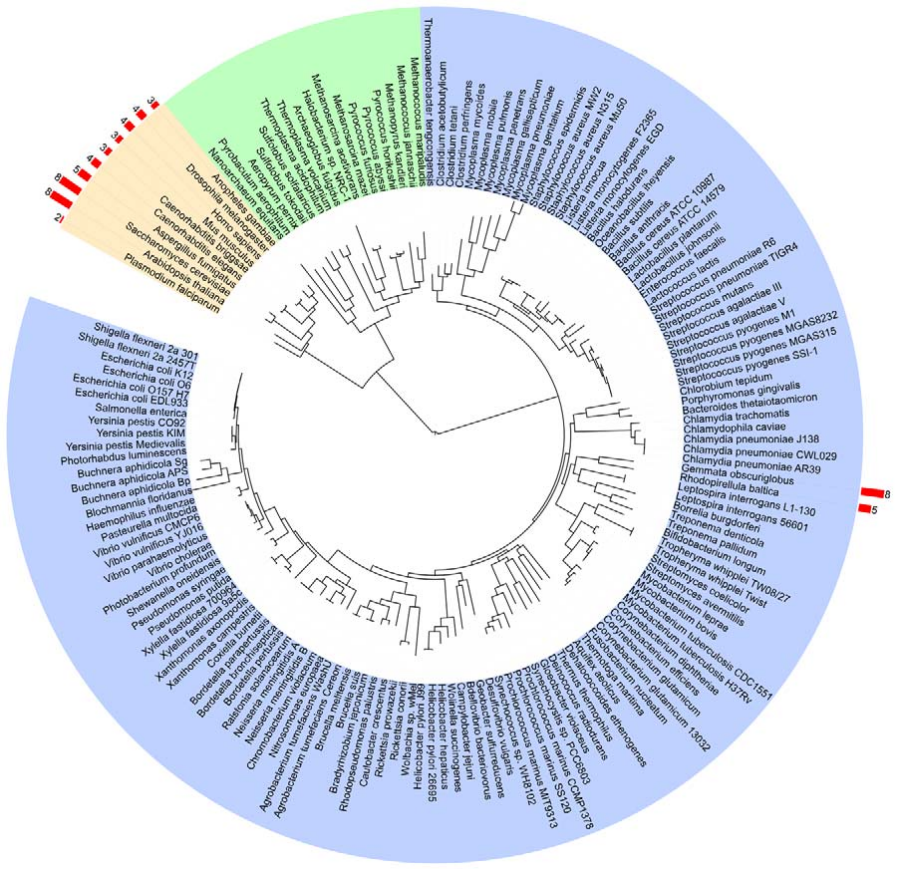
MC architecture detection. Global phylogeny of 212 organisms for which an alignment of 31 universal protein families could be built, adapted from [50], drawn with iTOL [51]. Eukaryotes, archaea, and eubacteria are grouped with orange, green, and blue backgrounds, respectively. The number of MC proteins found in each proteome is indicated on the external arc with red bars (see Supporting Information for the complete proteome dataset). Note that this tree includes only two members of the PVC superphylum (both are planctomycetes).

Thus, proteins predicted to have the MC architecture were detected in all eukaryotes, as expected—however, they were also unexpectedly detected in the proteomes of several members of the bacterial Planctomycetes-Verrucomicrobia-Chlamydiae (PVC) superphylum (Figure 1; Table 1; Tables S1 and S2). We found 11, 11, 8, and 5 genes coding for MC-like proteins in the Planctomycetes *B. marina*, *P. maris*, *G. obscuriglobus*, and *R. baltica* proteomes, respectively, and 16, 14, and 9 in the Verrucomicrobiae *V. spinosum*, *C. flavus*, and *P. parvula*, and 9 in the Lentisphaerae *L. araneosa*. We did not find MC-like protein coding genes in the Planctomycetes *C. Kuenenia stuttgartiensis*, in the Verrucomicrobiae *A. muciniphila*, *M. infernorum*, *O. bacterium*, and *O. terrae* or in the Lentisphaerae *V. vadensis* proteomes. Notably, we found no MC-like proteins in the Chlamydiae. Most of the sequences identified are annotated as uncharacterized or predicted proteins. All PVC MC-like proteins are derived from a single common ancestor, since they detect each other after a few rounds of PSI-Blast. Sequence-similarity based clustering of these sequences suggests that the most recent common ancestor of these organisms may have contained more than one such protein; all of the dendrograms obtained from these analyses contained several well-supported groups of sequences whose species composition is inconsistent with the presence of a single MC protein in the most recent common PVC ancestor (Figure S1).

**Figure 2:**
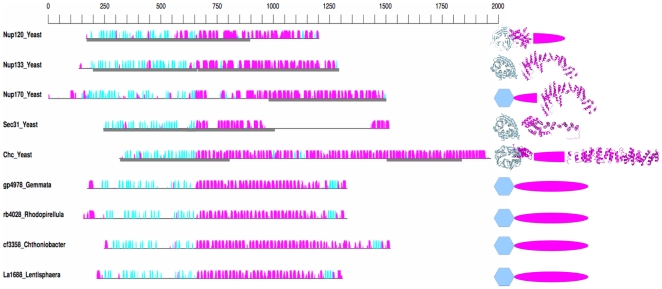
Secondary and tertiary structure of MC proteins. Representative yeast and PVC MCs are illustrated. Left: predicted secondary structure. The amino-acid scale is represented at the top. The black horizontal line represents the sequence of each MC protein. The predicted secondary structure [52], α-helices (magenta) and β -strands (cyan) are indicated by colored bars above each line. The height of the bars is proportional to the confidence of the predictions. When an atomic structure is available, the corresponding fragment is highlighted by a grey box below the sequence. Sequences are aligned around the transition from mainly β-sheet to mainly α-helical. Right: predicted and observed tertiary structure: Predicted fold types are represented by coloured shapes, cyan hexagon for β-propeller and magenta oval for SPAH domain. Where known, the atomic structure is represented with the same coloring scheme. PDB codes of the represented structures are 3hxr [8] and 3f7f [9], Nup120; 1xks [10] and 3i4r [12], Nup133; 3i5p [12], Nup170; 1bpo [53] and 1b89 [54], clathrin; and 2pm6 and 2pm9 [55], Sec31. Chc, clathrin heavy chain.

Sequence searches using PVC MC-like proteins as queries do not detect any sequences other than the PVC MC-like proteins, and such searches starting from the eukaryotic MCs do not detect any bacterial proteins, as reported previously [3]. These two facts demonstrate the necessity of using our structure-based search protocol. Despite the lack of significant sequence-similarity between eukaryotic and prokaryotic MCs, predicted secondary structure content and architecture (i.e., domain composition and organization) similarity links both sets of proteins at the structural level (Figure 2 and Figures S2–S9, Table 2), without implying homology (see Discussion).

### Planctomycete Compartmentalization

**Figure 3:**
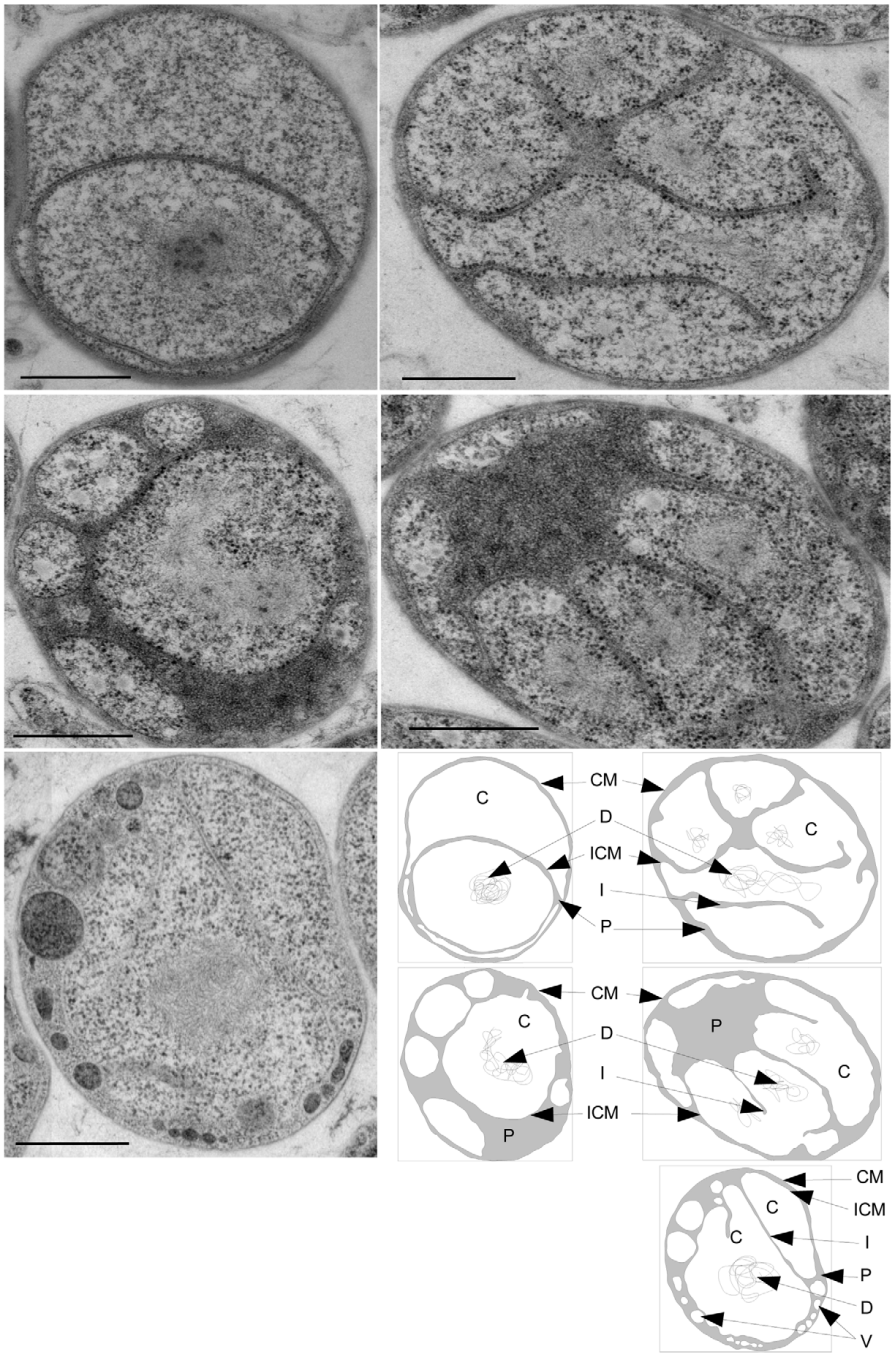
The *Gemmata* membrane morphology is variable. Electron micrographs of whole sectioned *G. obscuriglobus* cells representative of the morphologies observed. Lower right: schematic of the electron micrographs with the paryphoplasm colored in grey. CM, cytoplasmic membrane (+cell wall); ICM, intracytoplasmic membrane; P, paryphoplasm; I, invaginations of the ICM; D, DNA; V, vesicle. Scale bar: 500 nm.

The presence of proteins with the MC architecture in a bacterial phylum was unexpected [3],[13]. PVC is a monophyletic group whose members have dramatically different lifestyles and colonize a wide range of different habitats. However, they also have several unexpected similarities lending support to the monophyly of this supergroup [17],[18]. Unlike most other prokaryotes, members of the PVC superphylum have a compartmentalized cell plan [19],[20]. *G. osbcuriglobus*, a member of the Planctomycete phylum, is unique among prokaryotes in having cytoplasmic invaginations of the internal membrane that sometimes appear to surround the DNA with a double membrane envelope [19],[21]. Thus, we focused our analysis on *G. obscuriglobus*. To avoid artefacts related to sample fixation in conventional EM, we first investigated the membrane morphology in high-pressure frozen and freeze substituted *G. obscuriglobus* cells. We observed that the internal membrane morphology of *G. obscuriglobus* is variable and changes considerably during growth on solid culture medium. The main phenotypic observation is an irregular volume of the paryphoplasm, the space between the inner and outer membrane (Figure 3) [19]. In large colonies after 2 wk growth, the paryphoplasm can occupy up to 50% of the cell volume and frequently includes vesicle-like structures containing dark particles, most likely ribosomes. The content of the vesicles appears to have a different composition than the cytoplasm since it appears darker and denser in the electron micrographs (Figure 3), and the vesicle compartments are therefore presumably closed. The vesicles are unlikely to be artefactual as they were observed with two different fixation/substitution methods, osmium tetroxide-acetone and uranyl acetate-acetone, and have previously been reported using freeze fracturing [22].

**Figure 4:**
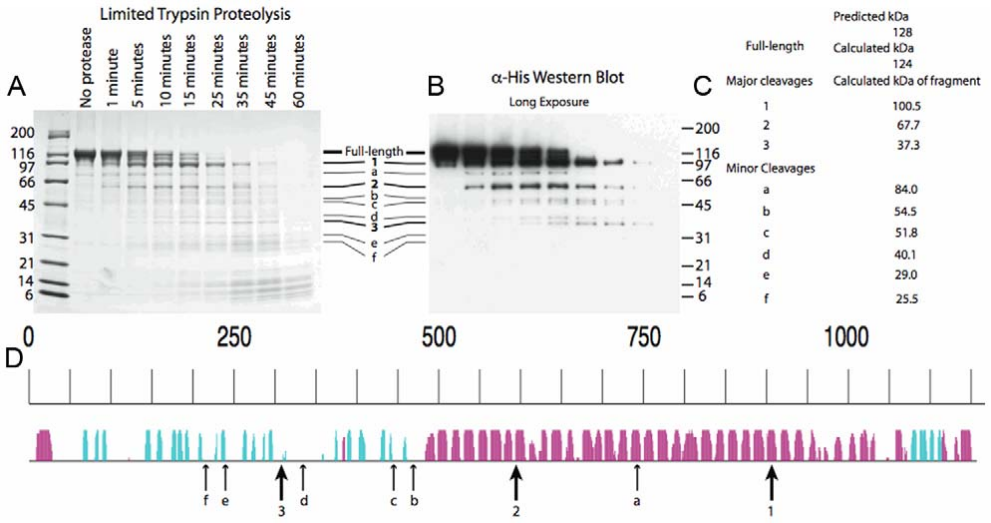
Limited proteolysis of gp4978. Purified N-terminal 10-His tagged gp4978 was trypsin digested and the reaction was stopped at various time points. The resulting fragments were electrophoretically separated. (A) Coomassie-stained SDS-page gel; (B) Anti-His antibodies stained Western blot; (C) Molecular weight of the resulting fragments (the full-length protein has a predicted weight of 128 kDa and a calculated one of 124 kDa); (D) Positions of cleavage are reported on the predicted secondary structure (Figure 2). The size of the arrow is relative to the susceptibility of the positions to cleavage.

To further localize one of the identified proteins, we cloned, overexpressed, and purified one of the *G. obscuriglobus* MC-like proteins, gp4978, in *Escherichia coli*. Limited proteolysis [23] supports the predicted MC architecture as protease-accessible sites are positioned similarly to those in eukaryotic MC proteins (Figure 4) [3],[13],[23],[24]. We then raised polyclonal antibodies against the gp4978 protein to investigate its localization in the cell. The antibodies recognized the gp4978 tagged protein in expressing *E. coli* cells but not in control extracts, indicating that it is specific for the protein (Figure S10). Western blot of *G. obscuriglobus* cell extracts indicated that the serum does not cross-react with other proteins, despite percentages of identity ranging from 22% to 28% between the *G. obscuriglobus* MC-like proteins. Additionally, we have characterized the specificity of the antibody using immuno-labeling. As limited labeling was observed outside the cell and pre-immune serum did not label the *G. obscuriglobus* cells, we concluded that the antibody is specific for gp4978. Labeling was not observed on control *E. coli* cells.

We performed a quantitative immuno-localization analysis on high-pressure frozen and freeze substituted *G. obscuriglobus* cells with affinity purified anti-gp4978 antibodies and secondary protein A-gold labeling. We initially analyzed cells with marked cytoplasmic membrane invaginations, most of which have paryphoplasm of considerable volume. In such cells, >95% of the antibody-gold particles localized in the paryphoplasm (*n* = 507). In *Gemmata* cells, labeling was not observed with two control sera, raised against human Mel-28 and *Aequorea victoria* green fluorescent protein, respectively.

**Figure 5:**
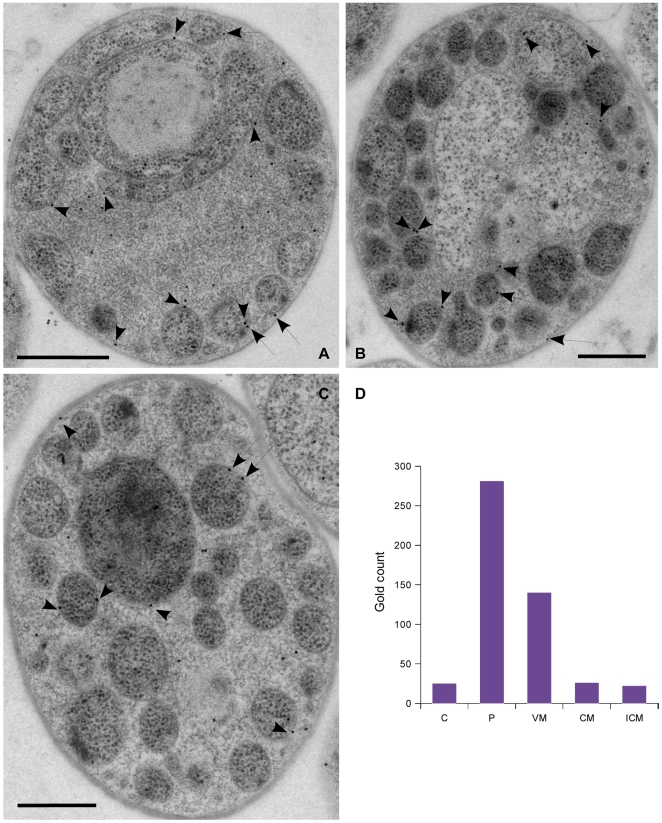
Sub-population of gp4978 associates with membranes. (A, B, and C) Electron micrographs of gp4978-immuno-labelled intra-paryphoplasmic vesicle-bearing *G. obscuriglobus* cells. Gold particles associated to membranes are indicated by arrows. Scale bars: 500 nm. (D) Chart of the distribution of 494 gold particles in the Cytoplasm (C), Paryphoplasm (P), Vesicle Membranes (VM), Cytoplasmic membrane (CM), and Intracytoplasmic membrane (ICM).

We then focused on cells with vesicles in the paryphoplasmic space. Most gp4978 either localized free in the paryphoplasm or in proximity to vesicle membranes (Figure 5). Fifty-nine percent of the gold particles were located in the paryphoplasm more than 10 nm from any membrane, and 28% were adjacent to the paryphoplasmic surface of a vesicle membrane. In addition, 5% were in contact with the outer membrane, 4% with the inner membrane, and 5% were located in the cytoplasm (*n* = 494 from four independent experiments). Thus, a significant fraction (>1/3^rd^) of the paryphoplasmic pool of gp4978 associates with intracytoplasmic membranes.

Eukaryotic MCs are in tight interaction with dynamic bent membranes [2]. Thus, the membrane localization of the *Gemmata* MC-like protein is similar to that of eukaryotic MCs. We therefore investigated the possibility of lateral gene transfer between a eukaryote and the bacteria by comparing the GC content and codon usage of the proteins and did not detect evidence of lateral gene transfer involving the planctomycete MC-like proteins. The codon usage and GC content of the MC-like protein genes is not significantly different from those of other planctomycete proteins, nor are they significantly similar to those of any proteins from other proteomes, including eukaryotes (Tables S3–S4).

## Discussion

We report the detection of proteins in the bacterial PVC superphylum displaying characteristics that were previously described only in components of the eukaryotic endomembrane system. Many members of the PVC superphylum have a compartmentalized cell plan, a feature normally associated with eukaryotic cells [20]. We report here that vesicles appear at a specific stage of the cell cycle in one of the PVC members, the planctomycete *G. obscuriglobus*, and that one MC-like protein localizes within close proximity to the membrane of those vesicles. The characterization of the other *G. obscuriglobus* MC-like proteins is ongoing.

### MC Architecture and Membrane Bending

The presence of MC-like proteins in one of the few known compartmentalized bacterial cells is striking. The fact that one such protein is found in proximity to intracellular membranes reinforces the importance of the MC protein architecture in the maintenance of compartmentalization, supporting the protocoatomer hypothesis and thus the fold assignments on which it is rooted [3],[13]. Strikingly, the individual MC folds, i.e., β-propellers and SPAH, dramatically increased in number with the emergence of eukaryotes [25],[26]. However, it is strictly the domain combination in this particular order that is uniquely associated with the eukaryotic and (as we now report) the PVC endomembrane system. There are no features of the combination of a β-propeller followed by a SPAH domain that obviously favors such a role. Both repeat domains have been proposed to be robust with respect to changes in their sequences, permitting their component repeats to rapidly lose their sequence similarity, allowing the protein to modify its function while retaining the core of its fold [27]. Indeed, despite their common ancestry [3],[4], the two coated vesicle MCs for which we have structural information, clathrin and Sec31, display drastic differences in tertiary structure, multimerization pattern, and cage formation. The clathrin and Sec31 β-propellers and SPAH domains are structurally divergent. In clathrin cages, the flexibility of the SPAH domains forming the edges of the cages and the flexibility of their interaction enable the formation of cages of various sizes [28]. In contrast, for COPII cages, it is the interaction angles between the β-propeller modules forming the vertex of the cages that accounts for most size variations [29]. This illustrates the multi-level diversity and extreme variation that two MC systems have achieved since their divergence from their last eukaryotic common ancestor, while retaining the core MC architecture.

### Nup or Coatomer?

MCs are part of two complexes in eukaryotes: nuclear pores and coated vesicles. Nuclear pore complexes bridge a double membrane, formed by a tightly bent single membrane, while vesicle coats surround a single membrane vesicle. gp4978 is unlikely to be a component of a nuclear pore-like structure in *G. obscuriglobus* as it is associated to a single membrane (Figure 5).

### MCs and Compartmentalization

Unlike most prokaryotes, most PVC members are compartmentalized cells [20]. We have detected MC-like proteins in one of the two Lentisphaera proteomes available, *Lentisphaera araneosa*, in which compartmentalization has been reported, but not in *Victivallis vadensis*. To our knowledge, compartmentalization has not been investigated in *V. vadensis*. The same observation applies to the Verrucomicrobia, where compartmentalization has been reported in the three species in which we detected MC-like proteins: *Chthoniobacter flavus*, *Pedosphaera parvula*, and *Verrucomicrobium spinosum* [20]. The genome of the 4^th^ Verrucomicrobium in which compartmentalization has been reported, *Prosthecobacter dejongeii*, is not available. We are not aware of analyses of the compartmentalized state of the Verrucomicrobia that we investigated and in which we did not detect MC-like proteins. In Chlamydiae, we analyzed the three complete proteomes available but did not detect any MC-like proteins. Again, no compartmentalization has yet been reported in Chlamydiae. The only Planctomycete in which we did not detect MC-like proteins and that is compartmentalized is the anammox *Kuenenia stuttgartiensis*. Although the absence of MC-like proteins could be the result of incomplete genomic information, the anammox exception might be related to the specific storage and containment function of this compartment. Anammox possesses unique features that differentiate it from other Planctomycetes [22], including the presence of ether-linked lipids. Thus, with the notable exception of the anammox *K. stuttgartiensis*, there is a correlation between the presence of MC-like proteins and compartmentalized cell state. This pattern indicates that the PVC last common ancestor already possessed MCs and was compartmentalized, as previously suggested [20]. PVC proteomes without MC-like protein genes probably represent cases of gene loss, as with the Chalmydiae, whose obligate intracellular parasitic lifestyle has resulted in massive gene losses [30],[31].

The protocoatomer hypothesis posits that a simple MC-containing coating module evolved in protoeukaryotes as a mechanism to bend membranes or stabilize bent ones [3]. The correlation between MCs and compartmentalization could be interpreted as supportive of the protocoatomer hypothesis but may of course also be due to convergent evolution.

### Convergent versus Divergent Evolution

Domain fusion/fission is known to have contributed to the birth of new proteins by the reshuffling of domain subunits [32]. Given the simplicity of the MC architecture and the large numbers of the two component domains individually found in most proteomes (Table S1), it is possible that both eukaryotic and bacterial MC proteins appeared separately, i.e., by convergent evolution. Indeed, no significant sequence similarity can be detected between the bacterial and eukaryotic MCs. Although this seems to indicate that the two sets of proteins are unrelated, it is noteworthy that sequence similarity is often lost during long periods of evolution (e.g., FtsZ and tubulin or MreB and actin). In fact, no sequence similarity can be detected between the eukaryotic MCs themselves, despite a common origin and significant structural similarity [4]. Thus, the absence of sequence similarity is uninformative concerning the origin of the two sets of proteins. On the opposite, the similarity of protein architecture is a first indication of a possible relationship between both sets, as convergence of fold architecture is a rare event [32],[33]. In addition, the similarity of localization, in close proximity to a variable membrane, is another argument in favor of a possible divergent evolutionary relationship between the eukaryotic and *G. obscuriglobus* MCs. Thus, the PVC MCs might be related to the eukaryotic ones, perhaps due to a lateral gene transfer event from eukaryotes. However, an analysis of the codon usage and GC content of the bacterial open reading frames did not detect any evidence of a recent lateral gene transfer.

### Implications of the MCs Detection in PVC

An autogenous origin for the eukaryotic endomembrane system was suggested more than 40 years ago [34],[35] and is supported by recent evidence [36],[37]. The apparent dearth of prokaryotic homologues to the endomembrane system [36] contrasts with the situation for mitochondria and chloroplasts, which are the result of endosymbiotic events. Morphological similarity between the planctomycete and the eukaryotic endomembrane systems was reported previously [19],[21]. The *G. obscuriglobus* inner envelope is topologically the closest bacterial analogue to the eukaryotic nuclear envelope, as it is a truly folded single membrane—an invagination of the intracytoplasmic membrane [38]. Others have analyzed the relationship between Planctomycetes and eukaryotes using sequence based searches with conflicting results [39]–[43]. This work represents, to our knowledge, the first analysis to use structural information to link the PVC superphylum and the eukaryotes. Our results present the molecular identification of such an intermediate between the eukaryotic and bacterial endomembrane systems, suggesting that the PVC bacterial superphylum contributed significantly to eukaryogenesis.

### Conclusion

This study describes the search for proteins that display what has so far been considered to be a typically eukaryotic architecture: the MC architecture. In eukaryotes, this architecture is restricted to proteins with a major role in compartment definition and maintenance, located in close contact with the endomembranes. We report the discovery of proteins with this architecture in the proteomes of compartmentalized bacteria from the PVC superphylum. One planctomycete protein was found to be located both in the paryphoplasm of the cells and associated with the membranes of paryphoplasmic vesicles. Our results demonstrate a previously unappreciated similarity between the compartmentalization machinery of prokaryotes and eukaryotes and thus suggest that the bacterial PVC superphylum contributed to the origin of the eukaryotic endomembrane.

## Material and Methods

### Bioinformatics

Complete proteome and genome sequences (as of November 2005) were initially downloaded from the CoGenT database [44]. The incomplete genome sequences for *G. obscuriglobus*, *Verrucomicrobium spinosum*, *Magnetospirullum magneticum*, and *Epulopiscium* sp. were obtained from The Institute for Genomic Research (www.tigr.org) and the EMBL-databank (www.ebi.ac.uk/genome/). Genomic data were translated in all six frames by the EMBOSS package software sixpack. Codon usages were obtained from GenBank, NCBI; Flat File Release 151.0. The analysis was updated in August 2009 to include recently sequenced genomes. Due to the particular cell plan observed in the Planctomycete/Verrucomicrobiae/Chlamydia/Lentisphaera superphylum [20], we included all proteins from this superphylum available from the Integrated Microbial Genomes database (http://img.jgi.doe.gov/) [45]. To limit the proteins to be screened to a manageable number, we restricted our analysis to proteins of size between 500 and 1,500 amino-acids.

### Domain Detection

We first searched all proteomes for proteins containing either one or both MC specific domains. Fold prediction for all sequences was performed by HHSearch [16], with default parameters using the October 2005 version of the SCOP70 database available from the HHSearch Web site. We considered a domain to be potentially present in a protein if the fold detection e-value was <1 over more than 40 positions. The resulting list was screened manually. All atom models were built and evaluated as previously described [3],[13]. A high concentration of these two domains in the gene pool was observed in many proteomes. We then searched for proteins that contain both MC domains. Although proteins composed of both β-propeller and SPAH domains can be found in most proteomes, these proteins form a higher fraction of the planctomycete protein sets. Finally, we screened for proteins with the MC architecture, and we required the β-propeller domain to be located N-terminal to the SPAH domain.

### On Domain Detection Sensitivity and Specificity

Although our single domain detection protocol almost certainly yields a number of false negatives, we expect this rate to be low as most of the eukaryotic proteins known to have this architecture were recovered. We made no effort to minimize the rate of false positive detection since our aim was to maximize detection sensitivity. We expect this rate to be similar or identical for all species, and there is no reason we are aware of to expect PVC proteins to give a higher false positive rate than proteins from other organisms.

### Generation of Genomic DNA

*G. obscuriglobus* were grown in liquid PYGV medium [46] to an OD ∼0.2 and cells were harvested by centrifugation. ∼100 µl of pelleted cells were lysed by adding 200 µl of breaking buffer (2% Triton X-100, 1% SDS, 100 mM NaCl, 10 mM Tris-Cl pH 8.0, and 1 mM EDTA), 200 µl of phenol/chloroform/isoamyl alcohol (25∶24∶1) (Sigma, P3803), and 200 µl of glass beads. This solution was vortexed rigorously for 3 min before adding 200 µl of TE (10 mM Tris-Cl pH 8.0+1 mM EDTA). This solution was centrifuged at 14,000 rpm for 5 min. The aqueous layer was transferred to a fresh tube. An equal volume of chloroform was added, briefly vortexed, and then spun at 14,000 rpm for 5 min. The aqueous layer was transferred to a fresh tube. One mL of 100% ethanol was added to the tube, mixed by inversion, and left for 30 min at –20°C. The sample was then spun at 14,000 rpm for 15 min at 4°C to pellet the DNA. The pellet was washed with 500 µl 70% ethanol and the tube was spun for an additional 5 min. After removing the ethanol the tube was left to dry at room temperature. Once dry, the pellet was resuspended in 200 µl of water.

### PCR Amplification of ORFs

To amplify *G. obscuriglobus* ORFs standard molecular biology protocols were used. Briefly, each ORF was amplified using AccuPrime Pfx DNA polymerase (Invitrogen, Carlsbad, CA) according to the manufacturer's specifications. One hundred ng of genomic DNA was used as a template in each reaction. Primers, synthesized by Integrated DNA Technologies, were designed to engineer a 5′ Nde I restriction and a 3′ Eco RI restriction site into each ORF for subcloning purposes. Primers used to amplify the gp4978 gene were:

5′gggattcccatatgcctcgctaccttctcgcattgccg and 5′gtcggaattcttattacttcttcaacgggtccttcaagctcgtcagg.

PCR products were run on a 1% agarose gel and bands of the expected sizes were excised from the gel, gel purified (MP Biomedicals, Geneclean II Kit), and then TOPO cloned into the pCR-Blunt II-TOPO vector (Invitrogen, Carlsbad, CA; K2800-20). Positive clones were first verified by restriction digest with Eco RI. Clones having the expected pattern of bands were sequenced using internal, gene-specific primers covering the entire ORF. A clone was identified in which no amino acid altering mutations were identified.

The ORF was then subcloned into the pSKB2-His10 bacterial expression vector using Nde I and Eco RI restriction enzymes. This introduced an N-terminal 10-Histidine tag for purification. Candidate inserts into the pSKB2-His10 plasmid were sequenced at both the 5′ and 3′ end of the ORF to ensure correct insertion into the plasmid, pSKB2-His10–ORF 4978.

### Recombinant Expression and Purification

Plasmids were transformed into *E. coli* BL21 (RIL) cells. Five ml overnight cultures were grown and used to inoculate 1 l of LB medium plus antibiotics (50 µg/ml kanamycin and 25 µg/ml chloramphenicol, final concentration). These 1 l cultures were grown at 30°C to an OD 0.6 at which time IPTG (final concentration of 1 mM) was added and the temperature was reduced to 25°C. Induced cultures were grown for 4 h before harvesting.

Six l of induced bacteria were harvested and resuspended in lysis buffer (20 mM HEPES pH 7.5 with 300 mM NaCl) with protease inhibitors and then lysed by microfluidization. The resulting lysate was spun at 20,000 rpm for 35 min in a Ti50.2 rotor to pellet the debris. The supernatant was collected and imidazole was added to a final concentration of 5 mM. The lysate was incubated with 8 ml of pre-washed TALON metal affinity resin (Clonetech, Mountain View, CA) for 4 h at 4°C. After incubation, the solution was poured over a column to collect the resin. The resin was then washed with 5 column volumes of lysis buffer, 20 column volumes of lysis buffer with 5 mM imidazole, and then 5 column volumes lysis buffer with 20 mM imidazole. Bound proteins were then eluted by passing 2 column volumes of elution buffer (20 mM HEPES pH 7.5, 300 mM NaCl, and 500 mM imidazole pH 8.0) over the resin. The eluate was then dialyzed extensively against lysis buffer to remove imidazole. After dialysis, the protein concentration was determined by Bradford assay, using BSA as a standard, and purity was assayed by SDS-PAGE electrophoresis.

### Limited Proteolysis

We slightly modified the previously described protocol [23]. One hundred µg of purified gp4978 was added to 900 µl of digest buffer (100 mM Tris-HCl (pH 8.5) with 0.01% SDS). Trypsin (11418025001; Roche Diagnostics, Indianapolis, IN) was added to give a weight ratio of 1∶200 of protease to the tagged protein. After protease addition, the sample was placed at 37°C and a 100 µl aliquot was removed at each time point. The sample from each time point was immediately TCA precipitated by adding 12 µl of 100% TCA. After centrifugation to pellet the protein fragments, samples were washed once in 90% acetone before being resuspended in SDS-PAGE sample buffer. Samples were run on 4%–20% Tris-glycine gels for Coomassie staining and Western blot analysis. In order to determine which gp4978 proteolytic fragments had an intact N-terminal 10-His tag, Western blot analysis using anti-His antibodies (Sigma Monoclonal anti-polyhistidine Product # H1029 at a 1∶3000 dilution) was performed.

### Antigen Injection and Affinity Purification

Purified antigen was injected into rabbits (Covance Immunology Services, Denver, PA) using the injection protocol described previously [47]. Each animal showed an excellent immune response to the injected antigen, and two production bleeds were performed before a final, terminal bleed.

For affinity purification, antisera from the terminal bleed of one rabbit was used. Affinity purification was performed as described previously [47]. After affinity purification, antibody elutions were concentrated and assayed by Western blot against whole cell lysates and purified recombinant protein.

### Electron Microscopy

*G. obscuriglobus* cells were grown for 8 d at 26°C on M1 agar plates [46] and either packed into capillary tubes or scraped from plates, placed in 0.1 µm Leica membrane carriers, and coated with hexadecane. Cells were then high-pressure frozen in a Leica EMPACT2 (Leica, Vienna) or HPM010 (Abra Fluids, Switzerland) high-pressure freezing machine. For morphological and immuno-labeling studies, cells were freeze-substituted and embedded as described in [48]. Thin sections (60 nm) were labeled with an anti-gp4978 antibody (1∶100) as described in [49]. Grids were imaged on a CM-120 (Biotwin) electron microscope.

## Supporting Information

Figure S1 **Sequence-similarity based clustering of PVC MCs.** Internal branches with greater than 70% bootstrap support are in red and are labeled with the number of 1,000 bootstrap datasets from which estimated dendrograms contained the branch. The scale bar indicates expected number of substitutions per alignment column. The tree is mid-point rooted to improve legibility-however, the tree should be considered as unrooted. The dendrogram was estimated from a trimmed gap-free alignment of 242 columns.
(6.02 MB TIF)

Figure S2 **Secondary structure predictions of the MC-like proteins detected in the** ***V. spinosum*** **proteome.** The amino-acid scale is represented at the top. The black horizontal line represents the sequence of each MC protein. The predicted secondary structure α-helices (magenta) and β-strands (cyan) are indicated by colored bars above each line. The height of the bars is proportional to the confidence of the predictions. Identifiers are from the IMG database (http://img.jgi.doe.gov/).
(1.51 MB TIF)

Figure S3 **Secondary structure predictions of the MC-like proteins detected in the** ***R. baltica*** **proteome.** Same convention as Figure S2.
(0.59 MB TIF)

Figure S4 **Secondary structure predictions of the MC-like proteins detected in the** ***P. parvula*** **proteome.** Same convention as Figure S2.
(0.91 MB TIF)

Figure S5 **Secondary structure predictions of the MC-like proteins detected in the** ***P. maris*** **proteome.** Same convention as Figure S2.
(1.48 MB TIF)

Figure S6 **Secondary structure predictions of the MC-like proteins detected in the** ***L. araneosa*** **proteome.** Same convention as Figure S2.
(0.92 MB TIF)

Figure S7 **Secondary structure predictions of the MC-like proteins detected in the** ***G. obscuriglobus*** **proteome.** Same convention as Figure S2.
(0.82 MB TIF)

Figure S8 **Secondary structure predictions of the MC-like proteins detected in the** ***C. flavus*** **proteome.** Same convention as Figure S2.
(1.80 MB TIF)

Figure S9 **Secondary structure predictions of the MC-like proteins detected in the** ***B. marina*** **proteome.** Same convention as Figure S2.
(1.24 MB TIF)

Figure S10 **gp4978 anti-serum Western blots.** Total cell extract, supernatant and pellet, and *E. coli* containing the empty expression vector or containing the poly-His gp4978 expression vector was probed with pre-immune (top) and anti-gp4978 (bottom) sera. Full-length gp4978 theoretical molecular weight is 124 kD. Two lower bands are observed both in *G. obscuriglobus* and in *E. coli* expressing lanes. Their size corresponds to the size of the two domain modules of gp4978 (b-propeller: 48 kD and SPAH: 76 kD). Mass-spectrometry confirmed that the lower bands in the *G. obscuriglobus* lanes are degradation products of the full-length protein gp4978.
(1.00 MB TIF)

Table S1 **β-propeller and SPAH domain proteins number in proteomes.** Number or proteins found in proteomes containing at least one β-propeller, one SPAH domain, both in any combination (bidomain), or with the MC architecture (Nt β-propeller followed by Ct SPAH). IncBacteria, incomplete Bacterial genome (as of November 2005).
(0.14 MB XLS)

Table S2 **Update of the Table S1 with a few selected proteomes (as of August 2009).** A, archaea; B, bacteria; E, eukaryotes; V, Verrucomicrobia; L, Lentisphaerae; P, Planctomycetes; C, Chlamydiae; D, draft; F, finished.
(0.11 MB XLS)

Table S3 **Codon usage tables and GC content for the proteins of** ***S. cerevisiae*** **,** ***C. trachomatis*** **,** ***E. coli*** **, and** ***G. obscuriglobus*** **compared to the ones of the** ***G. obscuriglobus*** **MCs.**
(0.11 MB XLS)

Table S4 **Codon usage RMSDs.** See Text S1.
(0.12 MB XLS)

Text S1 **Alignment of** ***G. obscuriglobus*** **proteins with structural template domains, phylogenetic analysis of the PVC MCs, and GC content and codon usage comparison.**
(0.02 MB RTF)

## Abbreviations

COP: coat protein complex
MC: membrane coat
PVC: Planctomycetes-Verrucomicrobia-Chlamydiae
SPAH: Stacked Pairs of α-Helices
