# Correction: The Compartmentalized Bacteria of the Planctomycetes-Verrucomicrobia-Chlamydiae Superphylum Have Membrane Coat-Like Proteins

**DOI:** 10.1371/journal.pbio.1002620

**Published:** 2018-02-14

**Authors:** Rachel Santarella-Mellwig, Josef Franke, Andreas Jaedicke, Mátyás Gorjánácz, Ulrike Bauer, Aidan Budd, Iain W. Mattaj, Damien P. Devos

There is an error in the fourth author's name. The correct author name in the byline should read: Mátyás Gorjánácz. The correct citation should be: Santarella-Mellwig R, Franke J, Jaedicke A, Gorjánácz M, Bauer U, Budd A, et al. (2010) The Compartmentalized Bacteria of the Planctomycetes-Verrucomicrobia-Chlamydiae Superphylum Have Membrane Coat-Like Proteins. PLoS Biol 8(1): e1000281. https://doi.org/10.1371/journal.pbio.1000281
