# Supplementary figures and images for: The Compartmentalized Bacteria of the Planctomycetes-Verrucomicrobia-Chlamydiae Superphylum Have Membrane Coat-Like Proteins
Source: PLoS Biol. 2010 Jan 19;8(1):e1000281. doi: 10.1371/journal.pbio.1000281 (PMC2799638; doi:10.1371/journal.pbio.1000281)

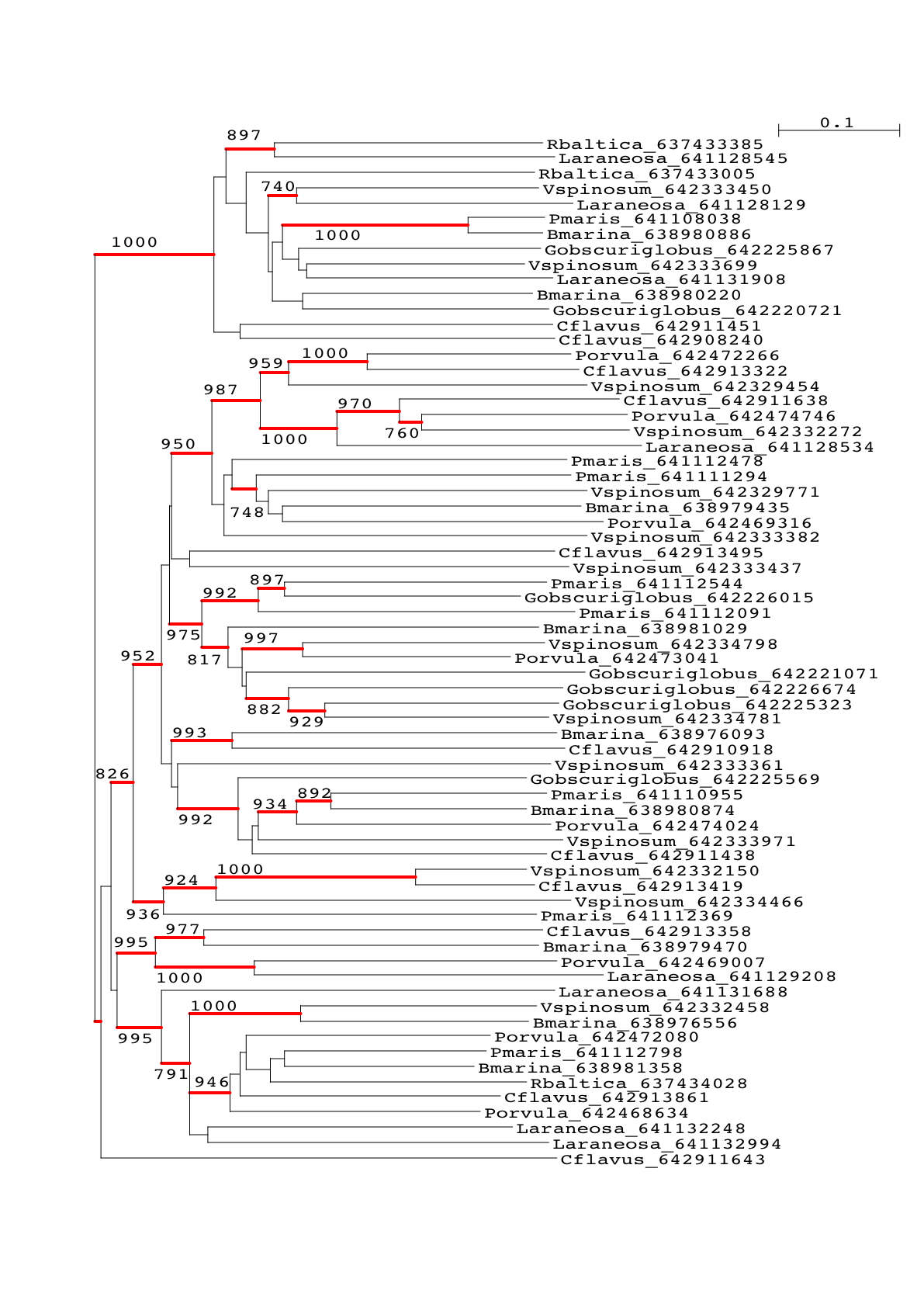

Supplement: Figure S1 — Sequence-similarity based clustering of PVC MCs. Internal branches with greater than 70% bootstrap support are in red and are labeled with the number of 1,000 bootstrap datasets from which estimated dendrograms contained the branch. The scale bar indicates expected number of substitutions per alignment column. The tree is mid-point rooted to improve legibility-however, the tree should be considered as unrooted. The dendrogram was estimated from a trimmed gap-free alignment of 242 columns. (6.02 MB TIF) [file pbio.1000281.s001.tif]

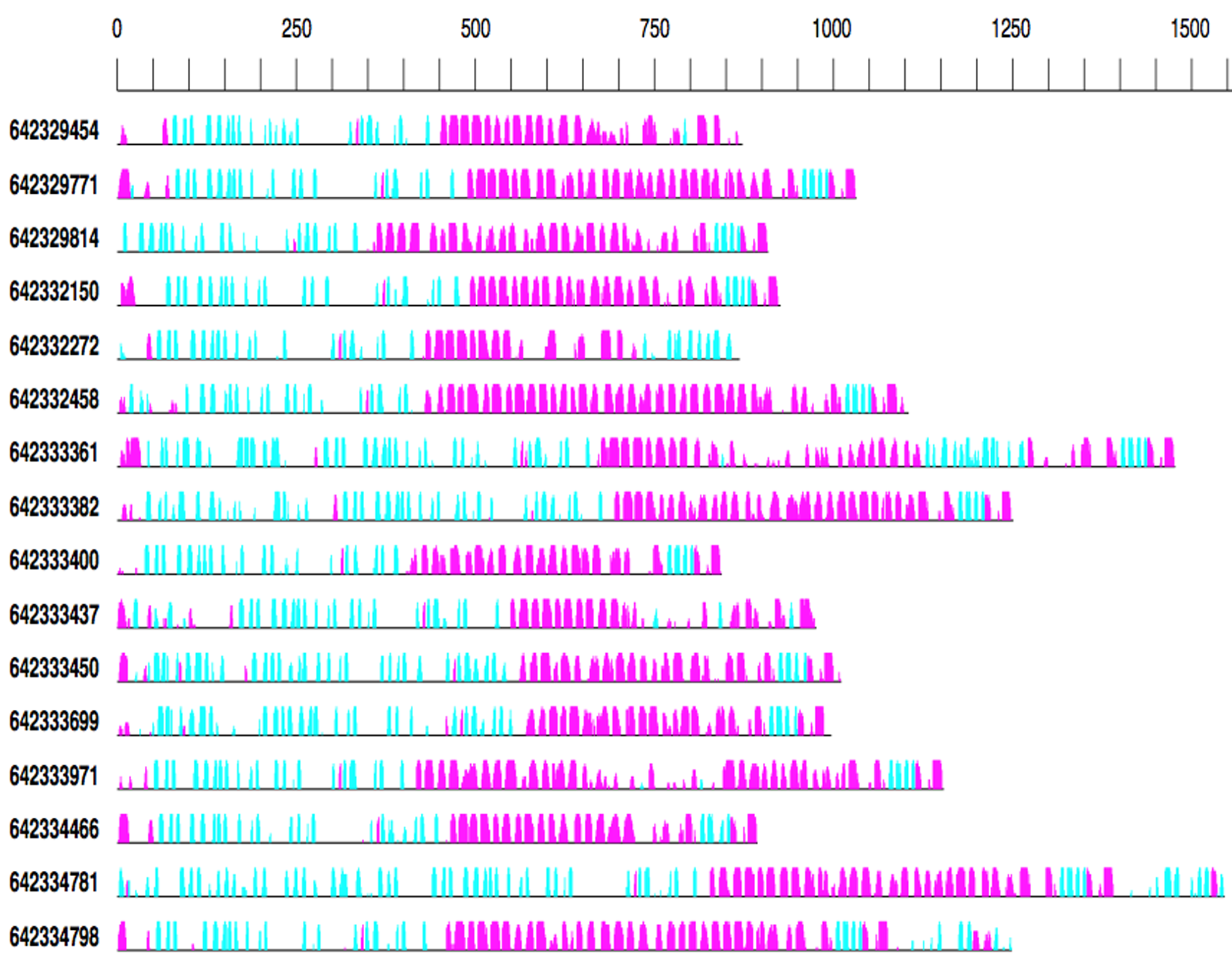

Supplement: Figure S2 — Secondary structure predictions of the MC-like proteins detected in the V. spinosum proteome. The amino-acid scale is represented at the top. The black horizontal line represents the sequence of each MC protein. The predicted secondary structure α-helices (magenta) and β-strands (cyan) are indicated by colored bars above each line. The height of the bars is proportional to the confidence of the predictions. Identifiers are from the IMG database (http://img.jgi.doe.gov/). (1.51 MB TIF) [file pbio.1000281.s002.tif]

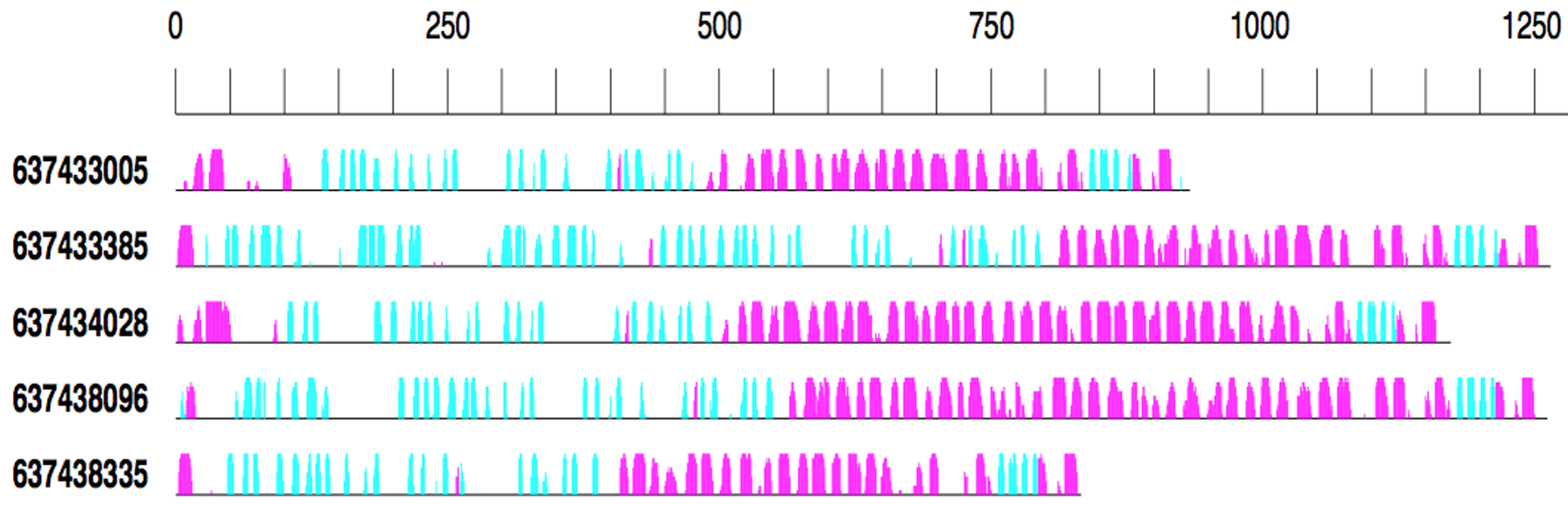

Supplement: Figure S3 — Secondary structure predictions of the MC-like proteins detected in the R. baltica proteome. Same convention as Figure S2. (0.59 MB TIF) [file pbio.1000281.s003.tif]

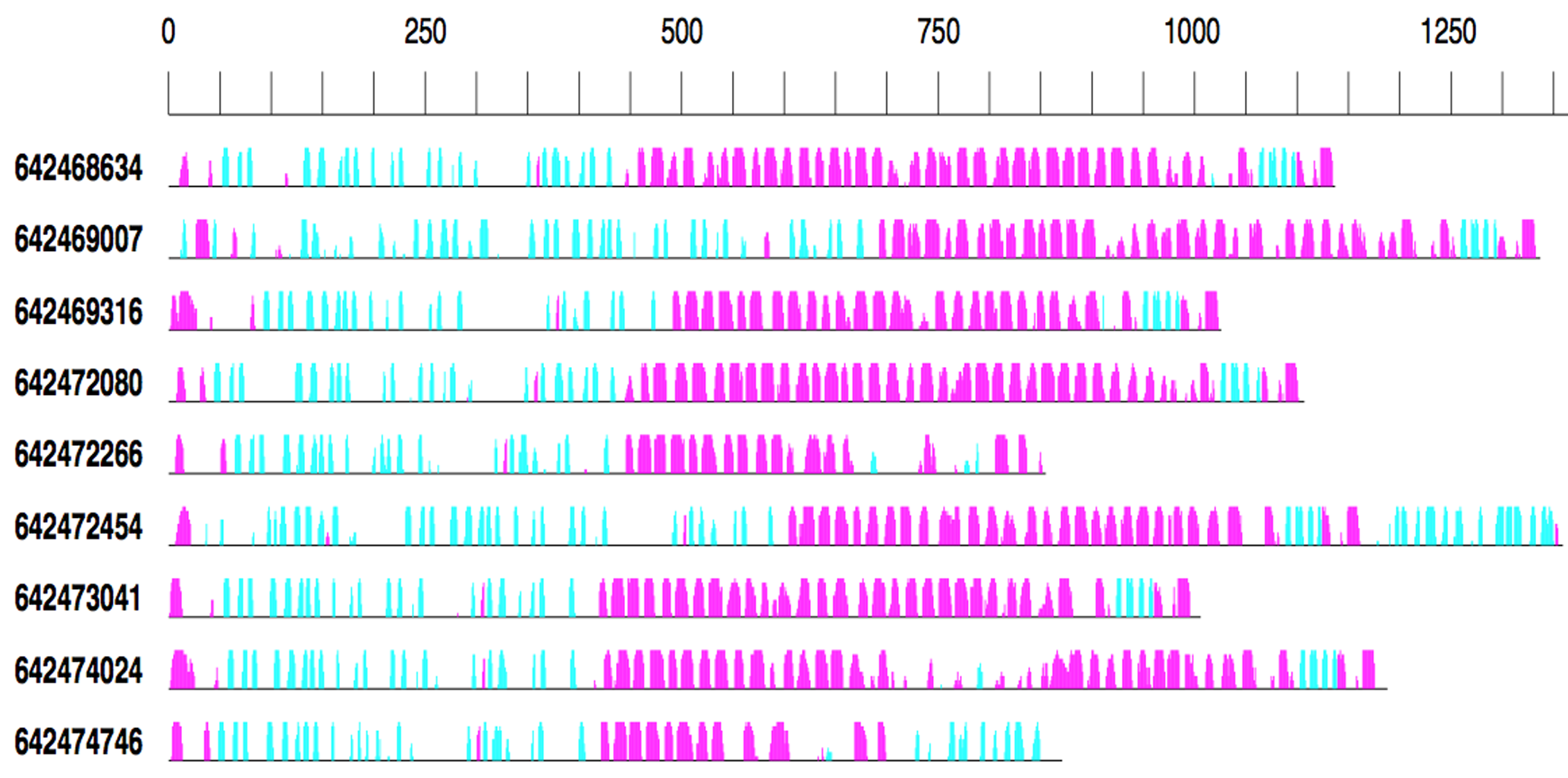

Supplement: Figure S4 — Secondary structure predictions of the MC-like proteins detected in the P. parvula proteome. Same convention as Figure S2. (0.91 MB TIF) [file pbio.1000281.s004.tif]

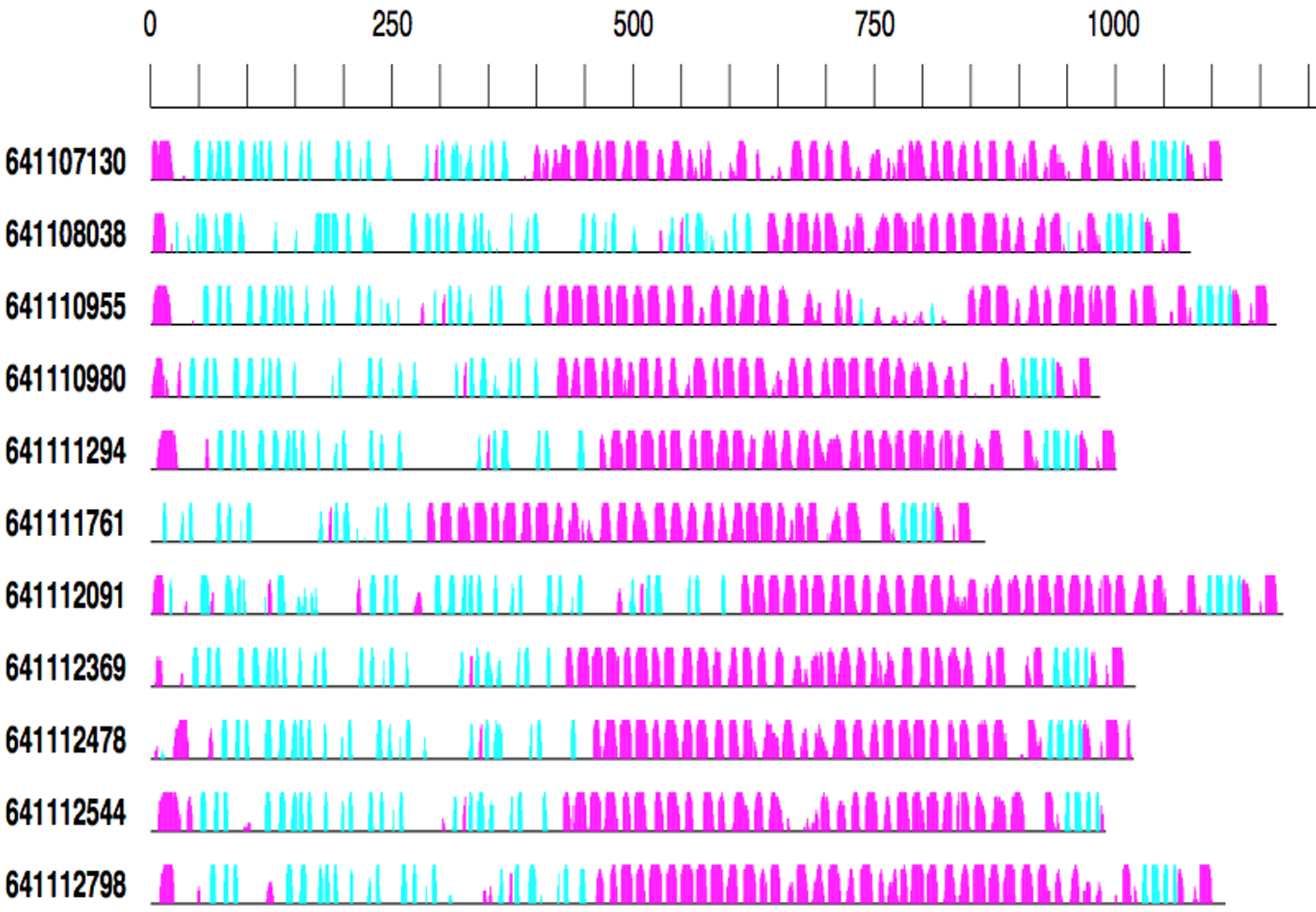

Supplement: Figure S5 — Secondary structure predictions of the MC-like proteins detected in the P. maris proteome. Same convention as Figure S2. (1.48 MB TIF) [file pbio.1000281.s005.tif]

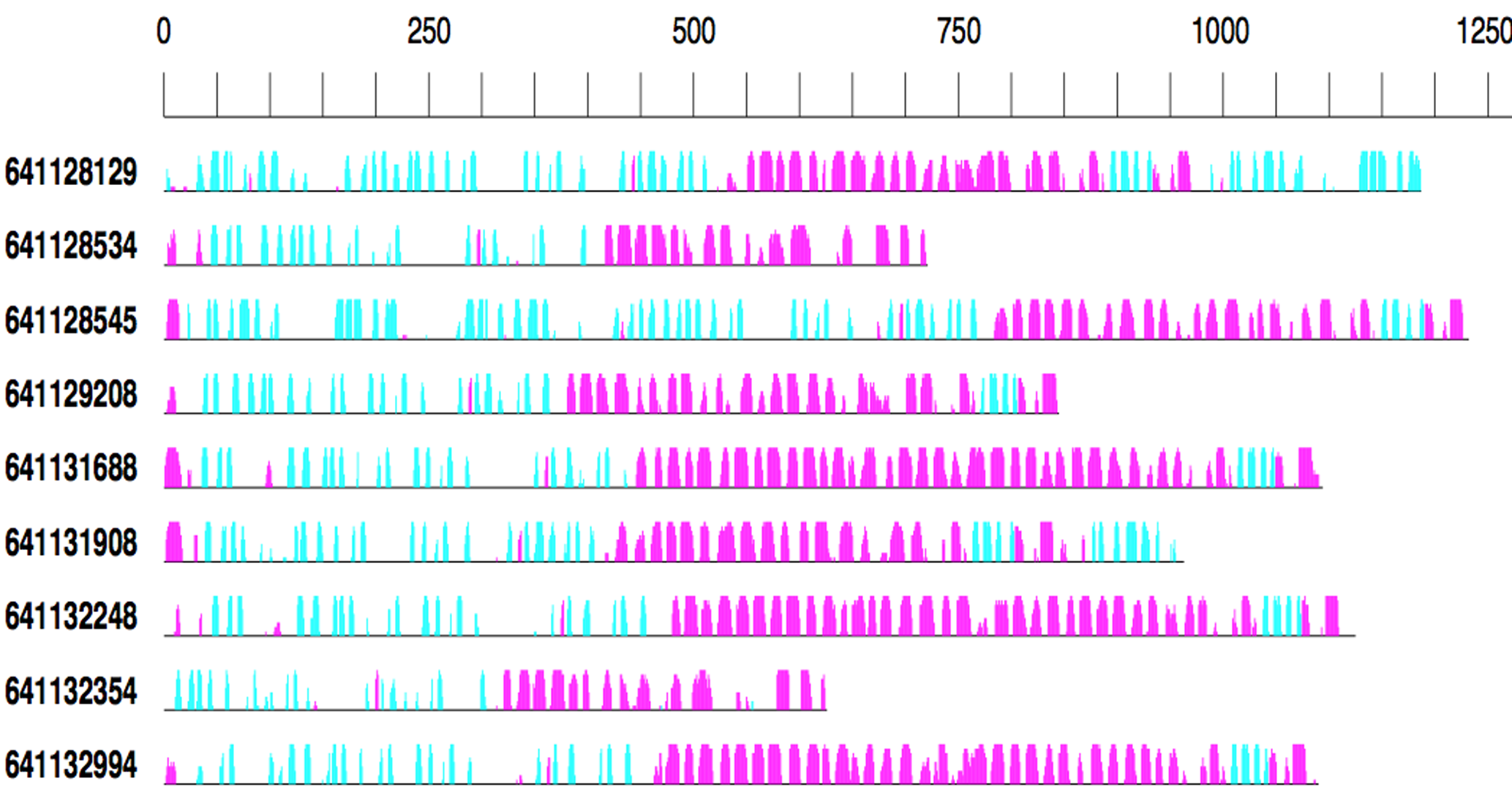

Supplement: Figure S6 — Secondary structure predictions of the MC-like proteins detected in the L. araneosa proteome. Same convention as Figure S2. (0.92 MB TIF) [file pbio.1000281.s006.tif]

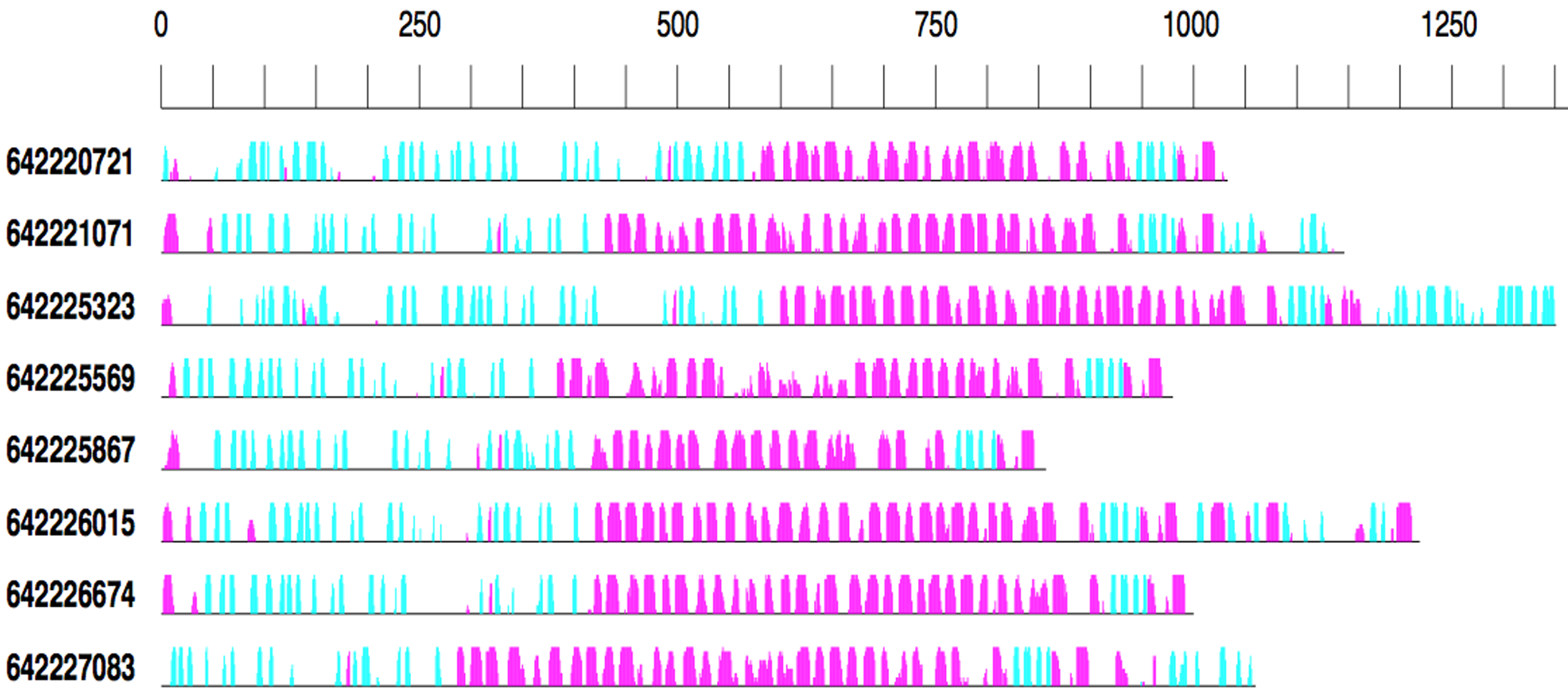

Supplement: Figure S7 — Secondary structure predictions of the MC-like proteins detected in the G. obscuriglobus proteome. Same convention as Figure S2. (0.82 MB TIF) [file pbio.1000281.s007.tif]

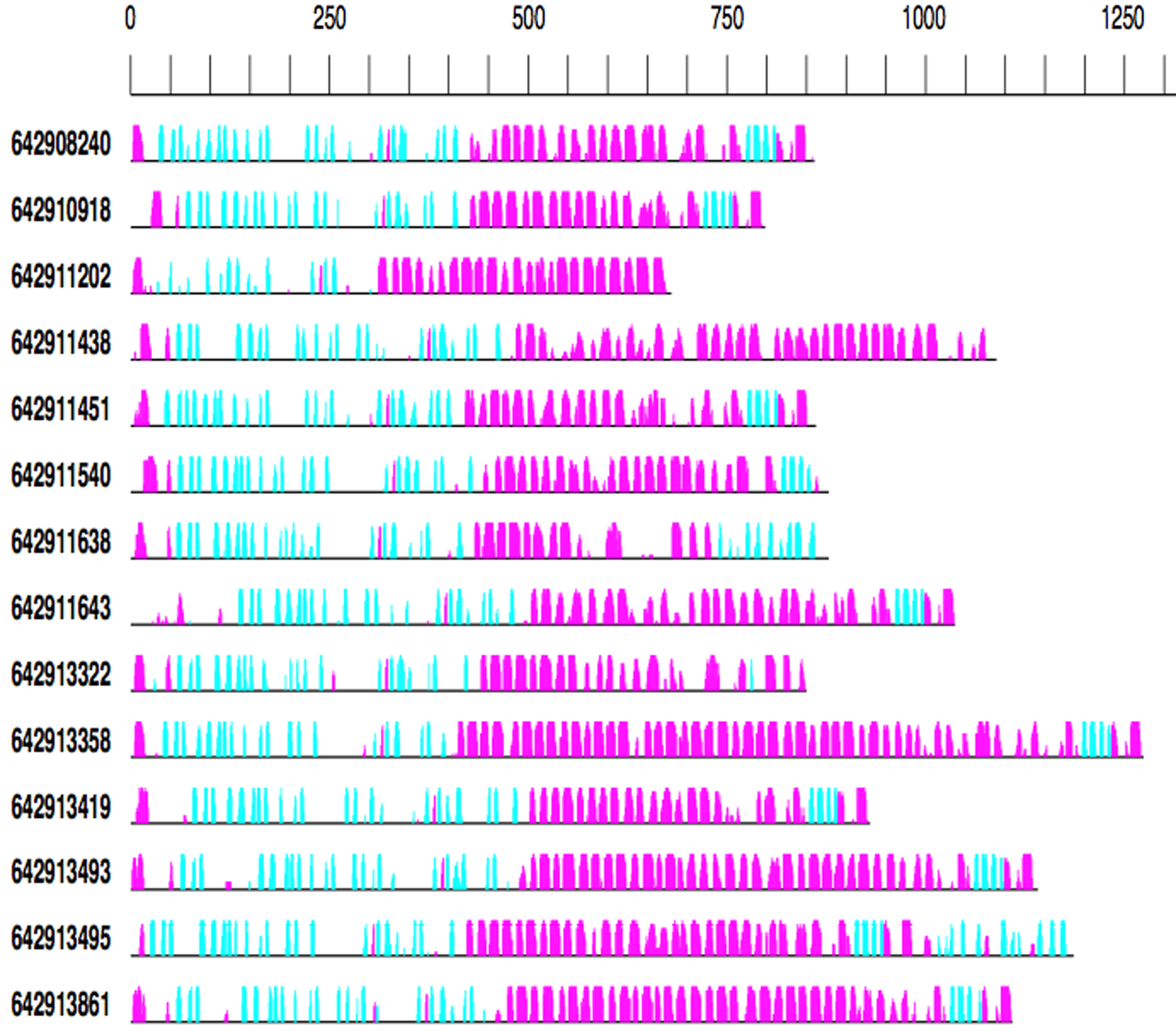

Supplement: Figure S8 — Secondary structure predictions of the MC-like proteins detected in the C. flavus proteome. Same convention as Figure S2. (1.80 MB TIF) [file pbio.1000281.s008.tif]

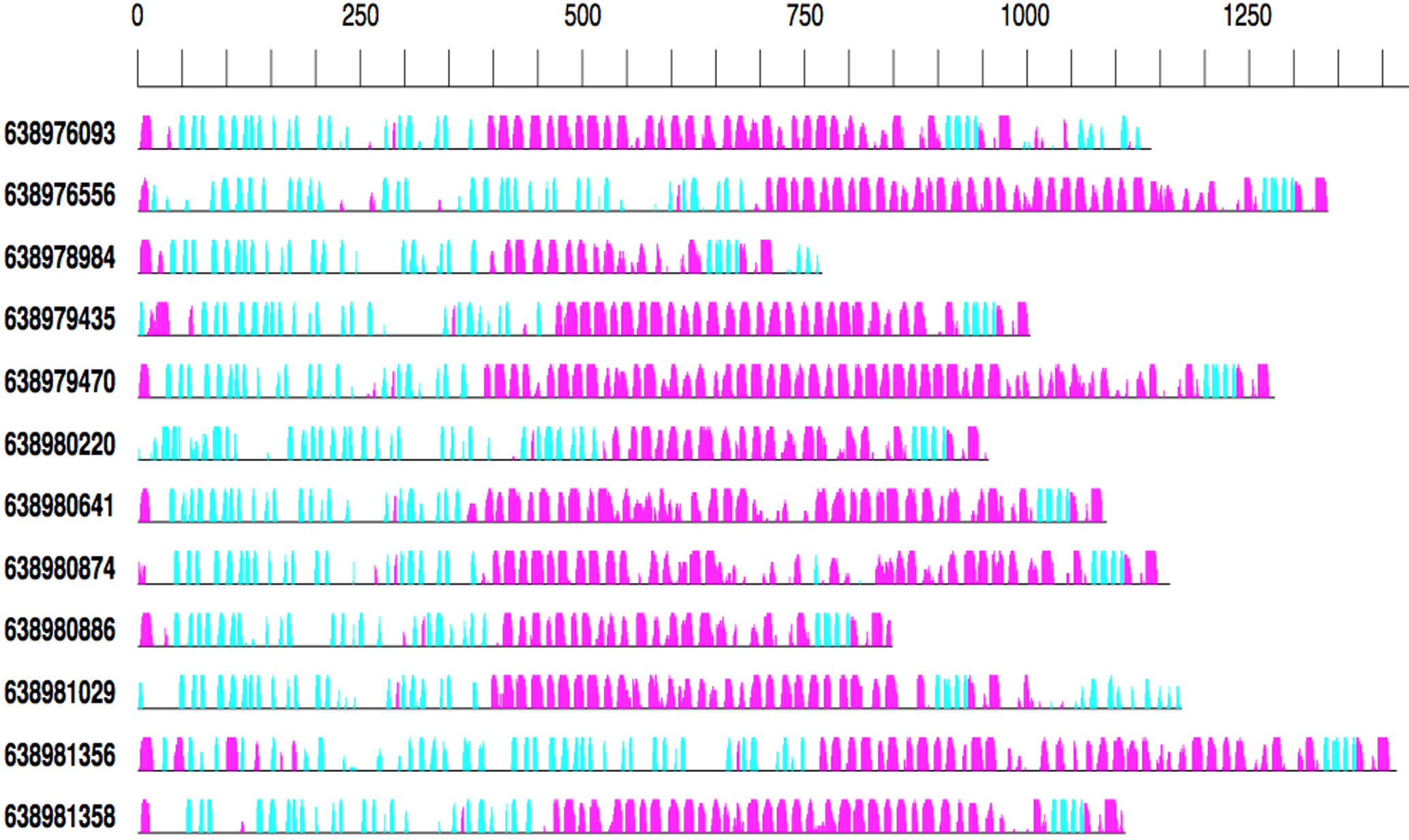

Supplement: Figure S9 — Secondary structure predictions of the MC-like proteins detected in the B. marina proteome. Same convention as Figure S2. (1.24 MB TIF) [file pbio.1000281.s009.tif]

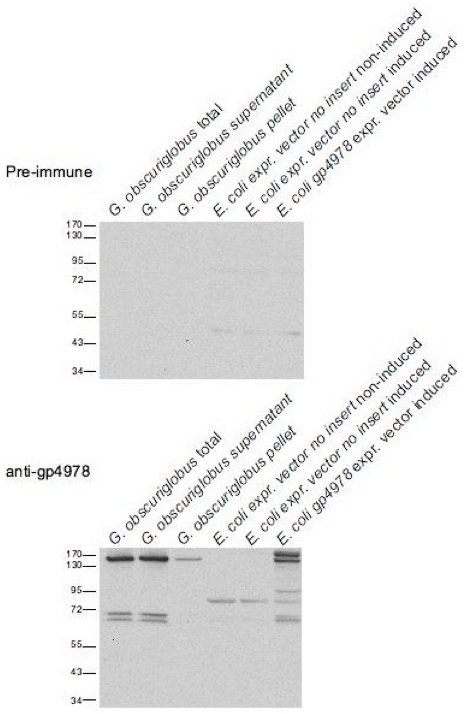

Supplement: Figure S10 — gp4978 anti-serum Western blots. Total cell extract, supernatant and pellet, and E. coli containing the empty expression vector or containing the poly-His gp4978 expression vector was probed with pre-immune (top) and anti-gp4978 (bottom) sera. Full-length gp4978 theoretical molecular weight is 124 kD. Two lower bands are observed both in G. obscuriglobus and in E. coli expressing lanes. Their size corresponds to the size of the two domain modules of gp4978 (b-propeller: 48 kD and SPAH: 76 kD). Mass-spectrometry confirmed that the lower bands in the G. obscuriglobus lanes are degradation products of the full-length protein gp4978. (1.00 MB TIF) [file pbio.1000281.s010.tif]
