# Supplementary material for: The Compartmentalized Bacteria of the Planctomycetes-Verrucomicrobia-Chlamydiae Superphylum Have Membrane Coat-Like Proteins
Source: PLoS Biol. 2010 Jan 19;8(1):e1000281. doi: 10.1371/journal.pbio.1000281 (PMC2799638; doi:10.1371/journal.pbio.1000281)
Supplement: Text S1 — Alignment of G. obscuriglobus proteins with structural template domains, phylogenetic analysis of the PVC MCs, and GC content and codon usage comparison. (0.02 MB RTF) [file pbio.1000281.s015.rtf]

Alignment of Gemmata proteins with structural template domains. Alignments were obtained from HHpred[1]⁠ and used to build an all atom model with Modeller[2].

>gp4978 residue 60 to 464
KAADKGLKVEVWASAPTMANPVSFCFDEKGKCYVAETTRFQVRVVWSSNGRGPADK-
---SEVFSGGYNRPQDGLAAGVLARK-----GSVYFT------------CIPDLYQLKDTNGDGKADEKKSLFTGFGPTVQFLGHDLHGLRMGPDGKLYF
SVGDRGFMVTTKEG---------------KKLEYPNTGAVLRCDPDGA-----------NLEVVHSGLRNPQEIAFDDFGNLFTYDNNCDSGDRARWVHI
VEGGDSGWRGGFQYSTGYHTPEVPQGNRG----------------------------------------AWNTEKLWHTQHEGQPAWIVPPLLHLGNGPA
GITHYPGIG-LNDKYKDHFFACDFTSSAGSSVIWAVSVKPKGASFEVQKPEPFLRGMVPTDCEFGPDG-AFYWSDWVGGWAPQNRGRIFRVTDA*
>1cru:  11:A: 435:A:b.68.2.1 (A:) Soluble quinoprotein glucose dehydrogenase:Acinetobacter calcoaceticus:1.50:0.17
KAKSENFDKKVILSN--LNKPHALLWGPDNQIWLTERATGKILRVNPESGSVKTVFQ
VPEIVNDADGQNGL-----LGFAFHPDFKNNPYIYISGTFKNPKSKELPNQTIIRRYTYNKSTDTLEKPVDLLAGLP---SSKDHQSGRLVIGPDQKIYY
TIGDQGRNQLAYLFLPNQAQHTPTQQELNGKDYHTYMGKVLRLNLDGSIPKDNPSFNGVVSHIYTLGHRNPQGLAFTPNGKLLQSEQGPNSDDEINL--I
VKGGNYGWPN-VAGYKDDSGYAYANYSAAANKSIKDLAQNGVKVAAGVPVTKESEWTGKNFVPPLKTLYTVQDTYNYNDPTCGEMTYICW----PTVAPS
SAYVYKGGKKAITGWENTLLVPSLKRGV----IFRIKLDPTYSTTYDDAVPMFKSNNRYRDVIASPDGNVLYVLTDTAG-------NVQKDDGS*

>gp4978 residue 682 to 958
GLVLALRKLKSEKVAEFVADADAKIVAEVARAAYDERIEGAIPVLAKLAEKSEPDAVAFRALAANYFLG----TPECAARVANFAARQSEPDYVRAFALK
LLGDWSKPPRRDPITGLTLDLAPRDTKIAAGALLKAGVAVFAGSTVVRSEAAQVAAKLNLKEFGPAMAAIVKDTKSPIATRVEALYAVDALKA--TEARD
LAAFALASDEPRLRAAGRSVKARLAPAEVLKELPALLENPNVSVAEKQGAFAILAAQKTSDAADQLLGLWLDRLNDAKVPGAL*
>1oyz:  15:A: 274:A:a.118.1.16 (A:) Hypothetical protein YibA:Escherichia coli:2.10:0.27
GLYNQCKKLNDDELFRLLDDHNSLKRISSARVLQLRGGQDAVRLAIEFCS-DKNYIRRDIGAFILGQIKICKKCEDNVFNILNNMALNDKSACVRATAIE
STAQRCKKNPIYS----------------PKIVEQSQITAFDKSTNVRRATAFAISVI---ATIPLLINLLKD--PNGDVRNWAAFAININKYDNSDIRD
CFVEMLQDKNEEVRIEAIIGLSYRKDKRVLSVLCDELKKNT----VYDDIIEAAGELGDKTLLPVLDTMLYKFDDNEIITSAI*


Phylogenetic analysis of the PVC MCs. Where MCs are found in a proteome, they are always present in multiple copies i.e. no proteome is found to contain only one such protein.

We investigated the source of the diversity of these proteins. In particular, we focused on the question of whether the most recent common ancestor of these organisms possessed multiple copies of such proteins.

Protein multiple sequence alignments (MSAs) were built using three different automatic MSA packages [3-5]. Sequence-similarity based clustering of the sequences calculated from different MSAs yielded different dendrograms. This is not surprising given the high divergence of the sequences from each other, the presence of repeats in the sequences, and the presence of several truncated sequences in the data set. However, these analyses consistently estimated dendrograms containing multiple clusters with low sampling error (as estimated using non-parameteric bootstrapping support of 70% or higher using 1000 bootstrap replicate datasets) that were incompatible with a scenario of evolution involving no lateral/horizontal gene transfer and a single copy of these proteins in the most recent common ancestor of the sampled organisms. For an example see figure S1, estimated from an MSA calculated using ProbCons[5]. These dendrograms provide relatively inaccurate estimates of the phylogeny of the sequences - however (i) as the sequences are in some cases very divergent from each other and (ii) due to the presence of several (artifactually or otherwise) truncated sequences in the alignment, any estimate of their phylogeny will be potentially relatively inaccurate. Thus, any conclusions made from this analysis are tentative.

Phylogenetic analysis methods.
We built automatic protein multiple sequence alignments (MSAs) for these proteins using MUSCLE v3.6 [3] using default parameters, MAFFT v6.240[4] with the maximum number of iterations set to 1000 and using the genafpair option, and ProbCons v1.12[5] using default parameters.
In all cases, some truncated sequences were excluded from the analyses following automatic alignment. Alignments were inspected using JalView v2.4 [6]. JalView was also used to select regions from the alignments that were reasonably well conserved in all sequences, and which contained no gaps. The resulting trimmed alignments were used to cluster sequences on the basis of pairwise identity-based distances, clustering using the neighbor-joining algorithm[7] estimating sampling error using non-parametric bootstrapping with 1000 replicates[8] as implemented by CLUSTALX[9]. The resulting dendrograms were inspected and prepared for the figure using NJPLOT[10].


Codon usage RMSDs. We compared the codon usages of a representative set of species from the 3 domains. The name of the species is orange, yellow and green for archeabacteria, eukaryotes and eubacterias, respectively. Codon usage and GC content were downloaded from the kazusa web site (). The codon usages were compared by calculating the pairwise RMSD according to the formula:

	RMSD = square root of ((sum Fci-Fcj)2/n)

where Fci is the is the frequency of the codon in genome I, Fcj is the frequency of the codon in the genome j and n is equal to 61 (64-3).

As an example, the RMSD between the Gemmata MCs and the proteins of E. coli, S. cerevisiae and G. obscuriglobus was of 27.3, 35.0 and 9.2, respectively, where the mean of all comparisons was of 25.68.


References

1.	Söding J (2005) Protein homology detection by HMM-HMM comparison. Bioinformatics 21: 951-60.

2.	Sali A, Blundell TL (1993) Comparative protein modelling by satisfaction of spatial restraints. J Mol Biol 234: 779-815.

3.	Edgar RC (2004) MUSCLE: a multiple sequence alignment method with reduced time and space complexity. BMC Bioinformatics 5: 113.

4.	Katoh K, Kuma K, Toh H, Miyata T (2005) MAFFT version 5: improvement in accuracy of multiple sequence alignment. Nucleic Acids Res 33: 511-518.

5.	Do CB, Mahabhashyam MSP, Brudno M, Batzoglou S (2005) ProbCons: Probabilistic consistency-based multiple sequence alignment. Genome Res 15: 330-340.

6.	Waterhouse AM, Procter JB, Martin DMA, Clamp M, Barton GJ (2009) Jalview Version 2--a multiple sequence alignment editor and analysis workbench. Bioinformatics 25: 1189-1191.

7.	Saitou N, Nei M (1987) The neighbor-joining method: a new method for reconstructing phylogenetic trees. Mol. Biol. Evol 4: 406-425.

8.	Felsenstein J (1985) Confidence limits on phylogenies: an approach using the bootstrap. Evolution 39: 783-91.

9.	Larkin MA, Blackshields G, Brown NP, Chenna R, McGettigan PA, et al. (2007) Clustal W and Clustal X version 2.0. Bioinformatics 23: 2947-2948.

10.	Perrière G, Gouy M (1996) WWW-query: an on-line retrieval system for biological sequence banks. Biochimie 78: 364-369.
